# Chaga Mushroom (*Inonotus obliquus*) Attenuates DNCB-Induced Atopic Dermatitis by Modulating Oxidative Stress and Cytokine Expression

**DOI:** 10.4014/jmb.2510.10032

**Published:** 2026-01-22

**Authors:** Junxiao Liu, Qun Zhang, Tianze Yang, Chang Liu, S. D. N. Kaushalya, Eun-kyung Kim, Yujiao Tang

**Affiliations:** 1 College of Pharmacy, Changchun University of Traditional Chinese Medicine, P. R. China; 2 College of Life Science, Changchun University of Science and Technology, P. R. China; 3 Department of Applied Bioscience, Graduate School of Dong-A University, Busan, Republic of Korea; 4 Nutritional Education Major, Graduate School of Education, Dong-A University, Busan, Republic of Korea

**Keywords:** Chaga mushroom extract, Atopic dermatitis, Oxidant stress, Inflammatory cytokines

## Abstract

Recent studies highlight the immunomodulatory properties of Chaga mushrooms. Atopic dermatitis (AD) is a multifactorial skin disorder involving interactions between innate and adaptive immune responses. This investigation evaluates the anti-atopic dermatitis activity of a by-product from ethanol-extracted Chaga mushroom (E-CME), positioning it as a sustainable natural candidate for AD therapeutic development. The antioxidant potential of E-CME was assessed using DPPH radical scavenging, H_2_O_2_ scavenging, metal chelation, and FRAP assays. *In vitro*, its immunomodulatory effects were evaluated in HaCaT and RBL-2H3 cell lines by measuring cytokine release and β-Hexosaminidase activity. For *in vivo* analysis, E-CME was topically applied to BALB/c mice sensitized with Dermatophagoides farinae extract (DFE), with AD induced by DNCB. Post-treatment, inflammatory cytokine expression and MAPK marker expression were examined. E-CME treatment significantly improved dermatitis scores (*p* < 0.05), mast cell infiltration, serum immunoglobulin levels (24.07% increase of IgG2, 26.19% decrease of IgE), oxidative stress markers, and skin cytokine gene expression. Spleen and lymph node weights, plus splenocyte viability, also improved with E-CME treatment. These findings suggest that E-CME possesses substantial therapeutic potential for AD management, attributed to its antioxidant and immunomodulatory effects, possibly mediated by the inhibition of oxidative stress-associated inflammatory pathways.

## Introduction

Medicinal mushrooms have long been utilized in traditional Oriental medicine, which significantly contributes to the development of nutritionally functional foods. *Inonotus obliquus* (*I. obliquus*), commonly known as the Chaga mushroom (CM), is a white rot fungus belonging to the family Hymenochaetaceae within the phylum Basidiomycota [1]. Due to its slow growth in alpine environments, often requiring more than a decade to mature, CM accumulates a high concentration of bioactive constituents, including betulin, polysaccharides, triterpenoids, flavonoids, sterols, polyphenols, and melanin, making it rare and pharmacologically valuable. Among these constituents, triterpenoids and polysaccharides exhibit significant immunomodulatory, while the polyphenols and flavonoids demonstrate potent antioxidant activity by protecting cellular components against oxidative damage induced by free radicals. CM extracts have also shown promising hypoglycemic effects. Furthermore, CM extracts have demonstrated anti-tumor, anti-inflammatory, and immune-stimulatory properties and antibacterial and antifungal actions [2, 3].

Atopic dermatitis (AD) is a chronic, complex, and currently incurable inflammatory disorder that impairs both the structural integrity and barrier function of the skin. Clinically, it is characterized by pruritus, dryness, erythema, weeping, and eczematous lesions [4]. Although its precise pathophysiology is not fully elucidated, AD is believed to result from a multifactorial interplay of genetic predisposition, environmental triggers, allergic sensitization, and microbial imbalances [5]. Current studies emphasize the role of both genetic and epigenetic mechanisms in AD, including altered DNA methylation and gene expression in keratinocytes and innate immune cells, which causes chronic inflammation [6]. Integration of barrier dysfunction and immune dysregulation hypotheses, now research informs with evidence that AD involves complex Th1, Th2, Th22, and Th17 immune pathway interactions and variable contributions based on the disease phenotype [7]. Compromise of the skin barrier leads to increased transepidermal water loss and promotes both T-helper 1 (Th1) and T-helper 2 (Th2)-mediated immune responses. During the acute phase of AD, Th2 cytokines—such as interleukin-4 (IL-4), IL-5, and IL-13—are upregulated, promoting class switching in B cells to produce immunoglobulin E (IgE). Hyperproduction of IgE is a hallmark feature of human AD [8, 9] and has also been well-documented in AD-like skin lesions observed in transgenic and NC/Nga mouse models [10, 11]. In contrast, chronic AD lesions are predominantly associated with Th1-mediated immune responses [12, 13]. This dysregulation of immune responses further exacerbates skin barrier dysfunction, perpetuating a vicious cycle of inflammation and barrier damage. Additionally, pro-inflammatory cytokines such as tumor necrosis factor-alpha (TNF-α) and interferon-gamma (IFN-γ) are implicated in both the acute and chronic phases of AD [4, 14].

Current therapeutic strategies primarily include the use of moisturizers and topical corticosteroids, which aim to restore skin hydration and reduce inflammation, respectively. Although corticosteroids remain an effective treatment option, their long-term use is limited by a range of adverse effects, including skin atrophy, acne, cataracts, growth retardation, cutaneous irritation, and potential hepatic and renal toxicity [15, 16]. Clinical guidelines advise against prolonged use of corticosteroids [17]. Natural products offer number of benefits, such as better patient compliance, lower cost, easier accessibility, and reduced side effects. Therefore, there is a growing need to explore novel natural compounds as potential alternative or complementary treatments for AD. Among these, CM (*Inonotus obliquus*) has attracted considerable attention for its anti-inflammatory and immunomodulatory properties [18, 19].

Although CM has been extensively studied for its anti-inflammatory and immune-enhancing effects, its therapeutic potential against AD remains poorly understood. Therefore, the present study investigates the anti-AD effects of topically applied Chaga mushroom extract (CME) in a murine model. Furthermore, the study aims to elucidate the underlying molecular mechanisms, thereby expanding the pharmacological understanding of CME in the management of inflammatory skin diseases.

## Materials and Methods

### Materials and Cell Culture

Mouse IgE and IgG2a enzyme-linked immunosorbent assay (ELISA) kits were purchased from Bethyl Laboratories (USA). Polymerase chain reaction (PCR) primers were obtained from Genotech (Republic of Korea). Antibodies for Western blot analysis, including those against transforming growth factor-β (TGF-β), TNF-α, inducible nitric oxide synthase (iNOS), and cyclooxygenase-2 (COX-2), were acquired from Cell Signaling Technology (USA).

All other chemical reagents, including calcium ionophore A23187, phorbol 12-myristate 13-acetate (PMA), p-nitrophenyl-N-acetyl-β-D-glucosaminide, hydroxyethyl piperazine ethane sulfonic acid (HEPES), L-glutamine, and dimethyl sulfoxide (DMSO), were purchased from Sigma-Aldrich (USA). TRIzol reagent for RNA extraction was sourced from Invitrogen (USA).

HaCaT and RBL-2H3 cell lines were obtained from the Korean Cell Line Bank (Republic of Korea). HaCaT cells were maintained in Dulbecco’s Modified Eagle Medium (DMEM), while RBL-2H3 cells were cultured in RPMI 1640 medium. Both media were supplemented with 10% fetal bovine serum (FBS) and 1% penicillin-streptomycin, and cells were incubated at 37°C in a humidified atmosphere with 5% CO_2_. Cell culture media and supplements were obtained from Gibco (USA).

For treatment experiments, HaCaT cells were seeded into 6-well plates at a density of 5 × 10^5^ cells per well and pre-treated with or without ethanol-extracted Chaga mushroom extract (E-CME) for 30 min. Prior to stimulation, cells were starved in serum-free DMEM for 12 h to synchronize the cell cycle and reduce baseline cytokine variation. Subsequently, cells were stimulated with a cytokine mixture containing IFN-γ (10 ng/ml) and TNF-α (10 ng/ml). After 6 h (for RNA extraction) or 15 min (for protein analysis), cells were harvested for downstream assays.

### Preparation of Extracts

Dried CMs were lyophilized and ground into a fine powder using a grinder. For ethanol extraction, 10 g of powder was mixed with 100 ml of 70% (v/v) ethanol (solid-to-solvent ratio, 1:10 g/ml) and extracted at 25 ± 2°C for 2 h with constant shaking (150 rpm); this procedure was repeated three times to ensure complete extraction. All extracts were filtered through 0.25 μm membranes and subsequently lyophilized over 3 days using a freeze dryer. Three independent batches were prepared under the same extraction protocol explained above and relative standard deviation was calculated as a measure of yield consistency.

### Determination of Betulinic Acid (BA) and Betulin in E-CME

The content of BA and and betuln in E-CME were determined using the reversed-phase high-performance liquid chromatography (RP-HPLC). A C18 analytical column was used, maintained at 30°C. The mobile phase consisted of methanol-water (87:13, v/v) delivered at a flow rate of 0.6 ml/min. The detection wavelength was set at 210 nm.

### Determination of Antioxidant Capacity

**Analysis of DPPH radical scavenging activity.** DPPH scavenging activity of various E-CME was measured according to a slightly modified method by Blois [20]. DPPH solution (1.5 × 10^-4^ M, 100 μl) was mixed with and without each extract (100 μl), after which the mixture was incubated at room temperature for 30 min. After standing for 30 min, absorbance was recorded at 540 nm by a microplate reader. Trolox was used as the positive control. The percentage of scavenging activity was calculated using the following equation:

Inhibition (%) = (Acontrol − Asample)/Acontrol * 100

where Acontrol is absorbance of reaction mixture without sample and Asample is absorbance of reaction mixture with sample at 540 nm.

Standard calibration curves were constructed by plotting the percent inhibition against that of Trolox. The sample inhibition percentage and the Trolox calibration curve gradient (x = [y – b] / a) ratio were determined as the Trolox equivalent antioxidant capacity (TEAC) and expressed in μM Trolox equivalents (TE/mg). The TEAC values of the samples were calculated using the following equation:

TEAC (μM TE/mg) = (Inhibition [%] – b) / a

**Hydrogen peroxide radical scavenging activity.** H_2_O_2_ scavenging activity was determined according to the method of Müller [21]. A 100 μl of 0.1 M phosphate buffer (pH 5.0) was mixed with each extract in a 96-microwell plate. 20 μl of hydrogen peroxide was added to the mixture, and then it was incubated at 37°C for 5 min. After the incubation, 30 μl of 1.25 mM ABTS and 30 μl of peroxidase (1 unit/ml) were added to the mixture, and then incubated at 37°C for 10 min.

The absorbance was recorded at 405 nm by a microplate reader, and the percentage of scavenging activity was calculated using the following equation:

Inhibition (%) = (Acontrol − Asample)/Acontrol * 100

where Acontrol is absorbance of reaction mixture without sample and Asample is absorbance of reaction mixture with sample at 405 nm.

Standard calibration curves were constructed, and TEAC values were determined as described in section DPPH.

**Metal chelating activity.** The chelation of Fe^2+^ by the test extracts was measured by the method of Carter [22]. Different concentrations of the test extracts were incubated with 0.05 ml FeCl_3_·6H_2_O (2 mM). The reaction was initiated by the addition of 0.2 ml ferrozine (5 mM). The total volume was made to 0.8 ml with methanol. Absorbance was read at 562 nm after 10 min. Sample with ethylenediamine tetra-acetic acid (EDTA) did not develop color, and served as the positive control, while the sample without EDTA served as the negative control. The metal chelation was calculated using the following equation:

Metal chelation (%) = (Acontrol − Asample)/Acontrol * 100

Standard calibration curves were constructed, and TEAC values were determined as described in section DPPH.

**Ferric reducing antioxidant power (FRAP).** FRAP assays were carried out according to the methods of Benzie and Strain [23]. A fresh working solution was prepared by mixing acetate buffer, 2,4,6-tri(2-pyridyl)-s-triazine (TPTZ) solution, and FeCl_3_·6H_2_O solution and then warmed to 37°C before use. Each extract was allowed to react with FRAP solution in a dark room at room temperature for 30 min. Readings of the colored product were then taken at 595 nm, and the percent scavenging activity was calculated using the following equation:

Inhibition (%) = (Acontrol − Asample)/Acontrol * 100

where Acontrol is the absorbance of the reaction mixture without the sample, and Asample is the absorbance of the reaction mixture with the sample at 595 nm.

Standard calibration curves were constructed, and TEAC values were determined as described in section DPPH.

### Cytotoxic Assessment Using MTT and β-Hexosaminidase Release Assay

**MTT Assay.** Cell viability was assessed using the MTT assay. Cells were treated with varying concentrations of E-CME for 24 h. The MTT solution was added and incubated for 4 h. Formazan crystals were solubilized in 100 μl of isopropanol (0.04 N HCl), and absorbance was measured at 570 nm [24]. All MTT assays were performed in triplicate (n = 3 independent experiments), and data are presented as mean ± standard error (SE).

**β-Hexosaminidase release.** RBL-2H3 cells were seeded in 6-well plates at 4 × 10^5^ cells per well and sensitized overnight with 800 ng/ml DNP-specific IgE. After washing with PBS, the cells were stimulated with DNP-BSA and incubated in extracellular buffer containing 0.1% BSA for 90 min. Alternatively, cells were pre-treated with E-CME for 1 h, then stimulated with 1 μM A23187 for 90 min. Supernatants were collected and incubated with 1 mM p-nitrophenyl-N-acetyl-β-D-glucosaminide in 0.1 M citrate buffer (pH 4.5) at 37°C for 90 min. Reactions were stopped with 0.2 M glycine buffer (pH 10.7), and absorbance was measured at 405 nm. This assay was repeated 3 times independently (n = 3), and statistical analysis was performed using one-way ANOVA with Tukey’s post hoc test.

β-Hexosaminidase release (%) = [A_medium / (A_lysate – A_medium)] × 100

### Animal Experiments

Eight-week-old female BALB/c mice (23 ± 3 g) were purchased from an approved laboratory animal supplier. In a 12-h dark cycle, the animals were housed with thermoregulation (22 ± 1°C) and humidity (40 ± 10%). Mice are acclimated to the laboratory environment for one week prior to the test. The animals are fed a standard rodent diet and receive free access to purified water (reverse osmosis autoclaved water). The mice were randomly allocated to five groups (n = 8 per group). Following is a list of the groups: Group I: control group - normal diet with water (CON); Group II: group-normal diet with E-MCE (100 mg/kg) (V); Group III: DNCB-induced group (AD); Group IV: DNCB-induced + E-CME (100 mg/kg) (AD+E-CME); Group V: DNCB-induced + Dermatop 0.25% (AD + Dermatop), (prednicarbate 2.5 mg/g). AD-like skin injuries were stimulated using 1% DNCB. The dorsal hair of the mice was shaved (approximately 2 × 3 cm) using an electric shaver before DNCB application. Then, the sensitization was performed with 200 μl of 1% DNCB, prepared by dissolving DNCB in an acetone–olive oil mixture (3:1 v/v). Four days after DNCB sensitization, the same volume (200 μl) of 0.5% DNCB was applied to challenge the dorsal skin in two-day intervals for three weeks. 200 μl of E-CME homogenized in the vehicle was topically applied to dorsal skin per day, three times a week for three consecutive weeks. Blood was collected for the next step of analysis. Mice were weighed, and mouse hearts were collected, frozen in liquid nitrogen, and stored at -80°C until further study, and heart tissues were also fixed in 10% formalin for the next histological analysis. Ethical approval for this study was obtained from “Changchun Sci-Tech University (CK2024).”

### Serum Biochemical Parameters

Serum samples were obtained by centrifuging mouse blood at 3,500 × g for 15 min at 4°C. The levels of immunoglobulin E (IgE) and immunoglobulin G2a (IgG2a) were quantified using ELISA kits (Bethyl Laboratories Inc., USA), in accordance with the manufacturer’s instructions. Superoxide dismutase (SOD) activity and malondialdehyde (MDA) concentrations were measured using commercial assay kits (Nanjing Jiancheng Institute of Biological Engineering, China), following the protocols provided by the manufacturer [31].

### PCR (qPCR)and Western Blotting

Total RNA was isolated from dorsal skin tissue using TRIzol reagent (Sigma-Aldrich), following the manufacturer’s protocol. qPCR analysis was performed as described in Section 2.4. The primer sequences used are listed in Table S1.

For protein analysis, total protein was extracted from skin tissue using cold radioimmunoprecipitation assay (RIPA) buffer. Lysates were clarified by centrifugation (three times at 18,894 × g for 20 min at 4°C). Protein concentrations were determined using a BCA protein assay kit (Thermo Scientific, USA). Western blotting was carried out following established procedures [25].

### Histological Observations

Excised ear tissues were fixed in 10% paraformaldehyde for 16 h, then embedded in paraffin. Randomly selected tissue sections from lesional areas, ensuring non-overlapping fields, were used for histological observations. Sections (6 μm thick) were stained with hematoxylin and eosin (H&E) for assessment of epidermal and dermal thickness under light microscopy. Mast cell infiltration was evaluated via toluidine blue staining. Mast cells were counted in five randomly selected fields per section.

### Data Analysis

All experimental results are presented as mean ± standard error (SE) from at least three independent replicates. Statistical analysis was conducted using the SAS software package (SAS Institute, USA). One-way analysis of variance (ANOVA) followed by Dunnett’s multiple range test was used to assess differences among groups. A *p*-value < 0.05 was considered statistically significant.

## Results

### Extraction Yield

The extraction process was done in three independent trials to determine the yield and the batch reproducibility. The extraction yield was 3.66 ± 0.14% for E-CME. Batch to batch variations in extraction yield are as presented in Table 2, which indicates a robust reproducibility of the extraction process under identical conditions. The relative standard deviation was 3.94% which demonstrates a higher yield consistency and batch reproducibility (< 15%).

### BA and Betulin in E-CME

**Fig. 1:**
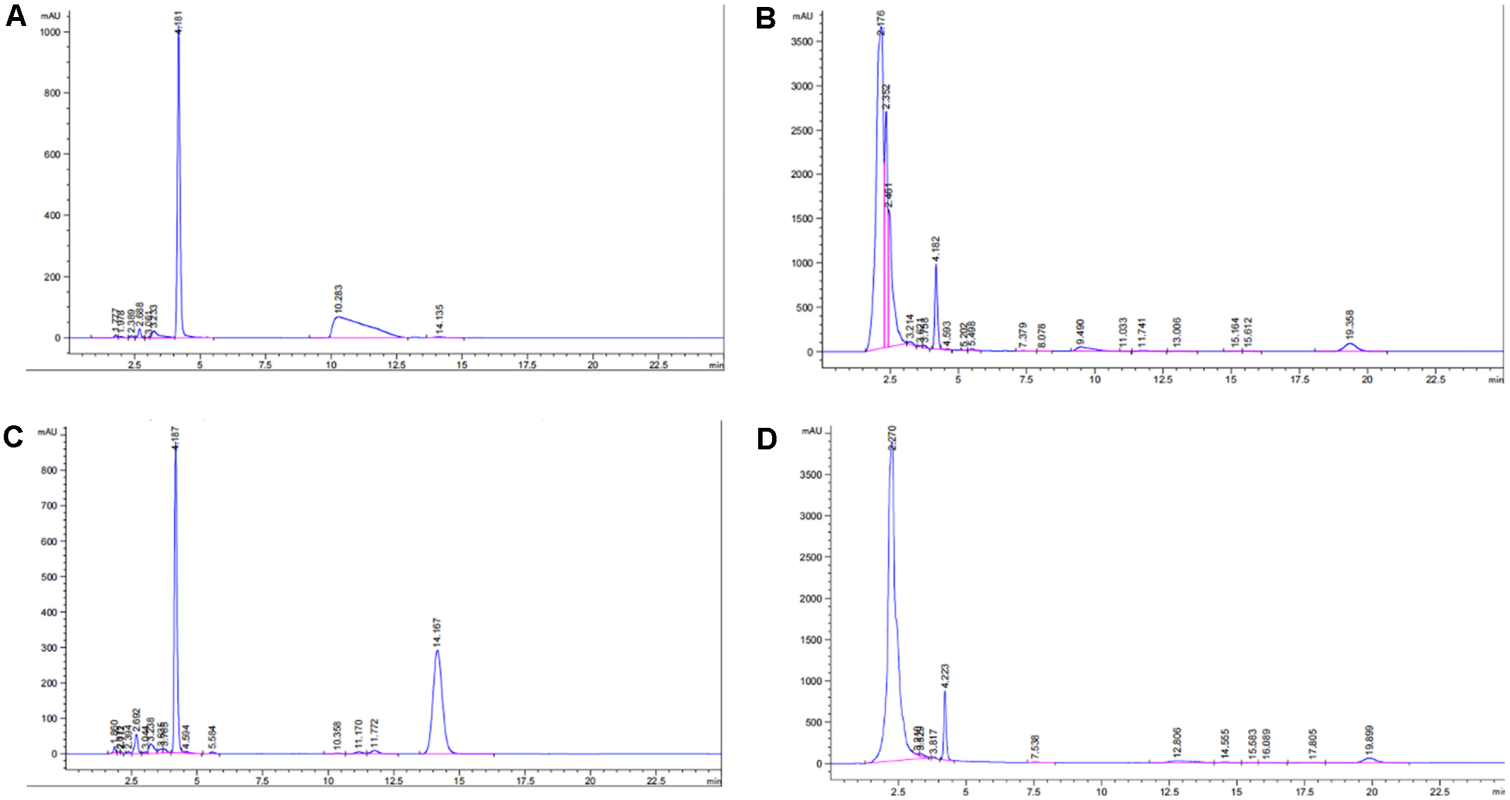
Representative reversed-phase high-performance liquid chromatography (RP-HPLC) chromatograms for the quantification of betulinic acid and botulin in E-CME. Chromatographic separation was performed using C18 analytical colums maintained at 30°C, with a mobile phase of methanol-water (87:13, v/v) delivered ata flow rate of 0.6 ml/min. detection was accomplished at 210 nm. (**A**) Chromatogram of betulinic acid standard, with the main peak observed in the range of 10.28–11.76 min, (**B**) Chromatogram of betulinic acid detected in E-CME, showing a peak at 11.741 min with a peaka rea of 261.471 mAU·s, (**C**) Chromatogram of botulin standard, with the main peak located in the range of 14.155–14.226 min. (**D**) Chromatogram of botulin detected in E-CME, showing a peak at 14.555 min with a peak area of 166.o mAU·s. These data confirm the presence and quantification of betulinic acid and botulin in E-CME under the described RP-HPLC conditions.

The composition of BA and betulin were assessed using RT-HPLC. The peak retention time of BA standard was observed in the range of 10.28 – 11.76 min under the described chromatographic conditions (Fig. 1A). Accordingly, a peak was observed at 11.741 min with a peak area of 261.471 mAU·s which corresponded to 35.341 μg of BA in 10 mg (3.4341 μg/g) of E-CME in E-CME chromatogram (Fig. 1B).

The retention time of the betulin standard peak ranged from 144.155 – 14.226 min (Fig. 1C) and the corresponding betulin curve was observed in the E-CME chromatogram at 14.555 min with a peak area of 166.0 mAU·s (Fig. 1D). As of that, 8.312 μg of betulin was found in 3.6 mg (2.309 μg/mg) of E-CME. The standard curves are provided as Fig. S1.

### Antioxidant, Cytoprotective, and Immunomodulatory Activities of Ethanol-Extracted Chaga Mushroom Extract (E-CME)

**Fig. 2:**
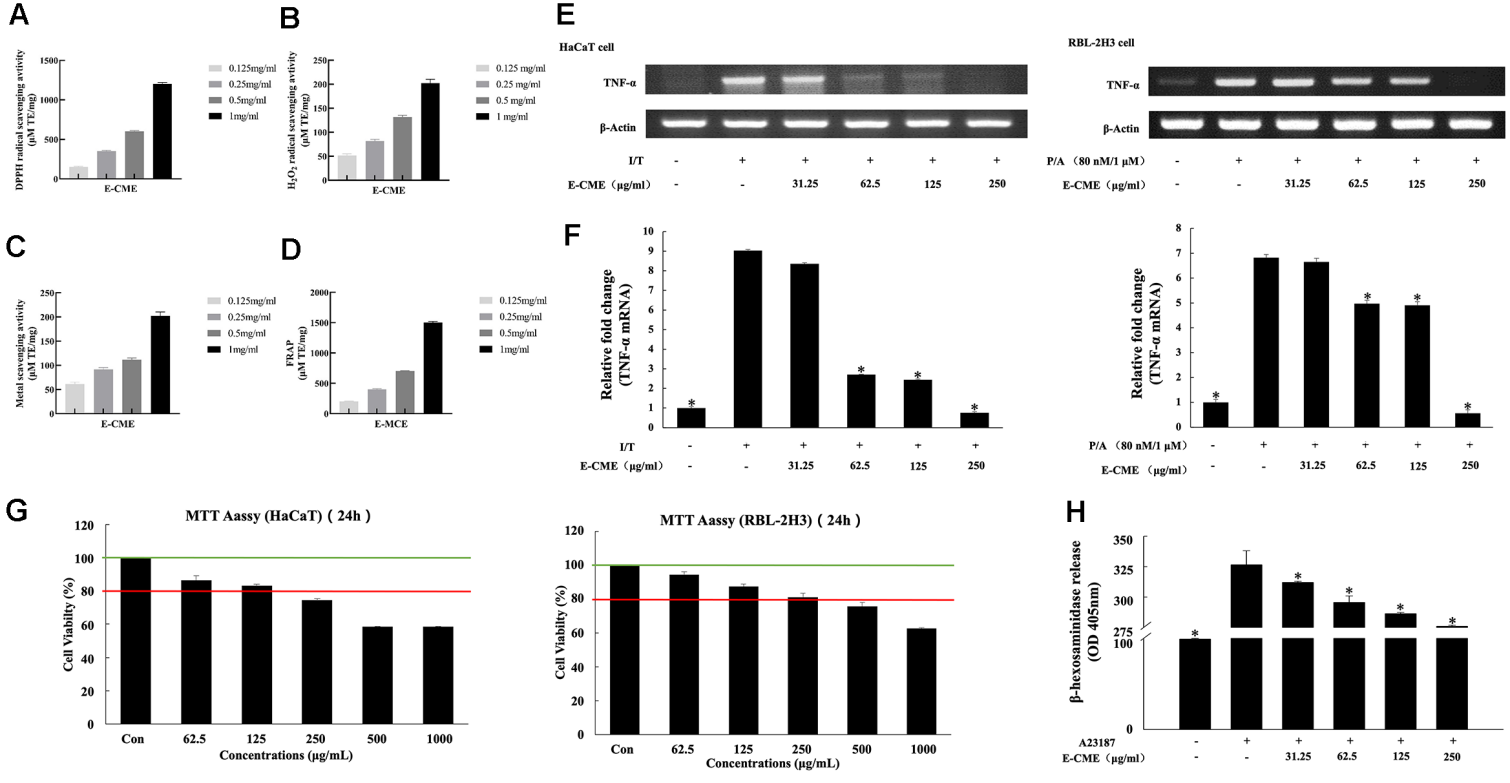
Antioxidant activity, cytotoxicity, TNF-α gene expression, and anti-allergic effects of ethanol-extracted Chaga mushroom extract (ECME). (**A–D**) *In vitro* antioxidant capacity of E-CME evaluated by (**A**) DPPH radical scavenging activity, (**B**) hydrogen peroxide (H_2_O_2_) scavenging activity, (**C**) ferrous ion (Fe^2+^) chelating activity, and (**D**) ferric reducing antioxidant power (FRAP) assays at concentrations of 0.125, 0.25, 0.5, and 1 mg/ml. Data are presented as mean ± SD (n = 3). Statistical comparisons were performed using one-way ANOVA with Tukey’s post hoc test; groups not sharing the same letter (a–c) are significantly different (*p* < 0.05). (**E–F**) Effects of E-CME on TNF-α mRNA expression in HaCaT keratinocytes (**E**) and RBL-2H3 mast cells (**F**). Cells were treated with various concentrations of E-CME, and TNF-α gene expression was analyzed by reverse transcription PCR using glyceradehyde 3-phospate dehydrogenase (GAPDH) as internal control. E-CME treatment downregulated TNF-α expression in a concentration dependent manner. Data represents SD from three independent experiments. Statistical significance was analyzed using one-way ANOVA followed by Tukey’s post hoc test (*p < 0.05). (**G**) Cell viability of HaCaT and RBL-2H3 cells following 24-h exposure to E-CME (62.5–1,000 μg/ml) determined by MTT assay. Viability remained above 80% at concentrations ≤125 μg/ml but declined significantly at higher concentrations. (**H**) Inhibitory effect of E-CME on β-hexosaminidase release in IgE-sensitized RBL-2H3 cells. Cells were presensitized with anti-dinitrophenyl (DNP) IgE and stimulated with DNP-bovine serum albumin (BSA). E-CME significantly suppressed β-hexosaminidase release in a dose-dependent manner, indicating inhibition of mast cell degranulation. Statistical significance was analyzed using one-way ANOVA followed by Tukey’s post hoc test (*p < 0.05).

The antioxidant capacity of E-CME was systematically evaluated using four *in vitro* assays: DPPH radical scavenging (Fig. 2A), hydrogen peroxide (H_2_O_2_) scavenging (Fig. 2B), ferrous ion (Fe^2+^) chelation (Fig. 2C), and ferric reducing antioxidant power (FRAP; Fig. 2D). In all assays, E-CME exhibited a clear dose-dependent increase in activity across the tested concentrations (0.125–1 mg/ml). Notably, its DPPH and FRAP activities were particularly prominent at 1 mg/ml, indicating substantial radical scavenging and electron-donating potential. Significant differences among concentrations were observed, as indicated by differing statistical groupings (*p* < 0.05), underscoring the extract’s potent redox-modulating capabilities. The standard curves are represented as Fig. S2.

To assess cytotoxicity and define a safe working range for biological applications, MTT assays were conducted in HaCaT keratinocytes and RBL-2H3 mast cells following 24 h treatment with E-CME (62.5–1,000 μg/ml). As shown in Fig. 2G (HaCaT) and 2F (RBL-2H3), E-CME maintained cell viability above 80% at concentrations ≤125 μg/ml. However, a significant decline in viability was observed at higher doses, particularly ≥500 μg/ml (*p* < 0.05), indicating concentration-dependent cytotoxicity. Based on these results, concentrations up to 250 μg/ml were selected for downstream anti-inflammatory investigations.

The anti-inflammatory properties of E-CME were evaluated by examining its effect on TNF-α mRNA expression in TNF-α and IFN-γ (T/I)-stimulated HaCaT keratinocytes and RBL-2H3 mast cells via reverse transcription PCR (Fig. 2E and 2F, upper panels). Densitometric analysis (Fig. 2E and 2F, lower panels) demonstrated that E-CME significantly downregulated TNF-α expression in a concentration-dependent manner in both cell lines, with maximal suppression observed at 250 μg/ml. These findings suggest that E-CME interferes with pro-inflammatory cytokine signaling at the transcriptional level.

Furthermore, anti-allergic effect of E-CME was assessed using a β-hexosaminidase release assay in IgE-sensitized RBL-2H3 cells stimulated with DNP-BSA. As shown in Fig. 2H, E-CME treatment resulted in significant, dose-dependent inhibition of β-hexosaminidase release, with vs of approximately 13%, 24%, 35%, and 47% at 31.25, 62.5, 125, and 250 μg/ml, respectively (*p* < 0.05). These results indicate effective attenuation of mast cell degranulation and allergic response.

Taken together, these findings demonstrate that E-CME exerts multifaceted bioactivities, including robust antioxidant effects, acceptable cytocompatibility at moderate concentrations, and significant anti-inflammatory and anti-allergic activities, thus supporting its potential application as a therapeutic agent in oxidative stress and immune-related skin disorders.

### E-CME Alleviates DNCB-Induced Atopic Dermatitis and Preserves Systemic Health in BALB/c Mice

To investigate the *in vivo* efficacy of E-CME, a DNCB-induced AD model was established in BALB/c mice. Mice treated with DNCB developed pronounced AD-like phenotypes, including erythema, dryness, excoriation, and lichenification. Topical application of E-CME or the positive control, Dermatop, was initiated following disease induction and continued for three weeks. Topical administration of E-CME was well tolerated throughout the experimental period, with no observable signs of erythema, edema, or irritation in areas outside DNCB-induced lesions, indicating good dermal compatibility independent of AD severity scoring.

**Fig. 3:**
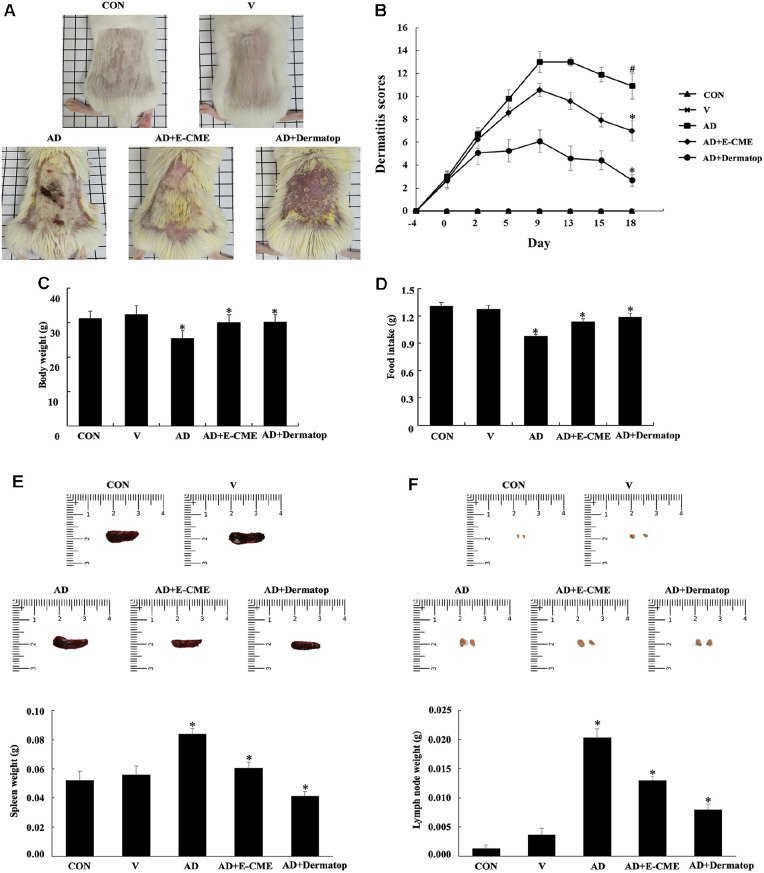
Therapeutic effects of ethanol-extracted Chaga mushroom extract (E-CME) on DNCB-induced atopic dermatitis (AD)-like symptoms, physiological status, and immune organ hypertrophy in BALB/c mice. (**A–D**) Evaluation of AD-like skin lesions and general physiological status. (**A**) Representative dorsal skin images from each group: normal control (CON), vehicle (**V**), DNCB-induced AD (AD), E-CME-treated AD (AD + E-CME), and positive control (A), groups demonstrating differences in erythema, dryness, and epidermal thickening. (**B**) Dermatitis severity scores were determined based on five clinical parameters: erythema/hemorrhage, pruritus/dryness, edema/excoriation, erosion, and lichenification, graded on a 0–3 scale (0 = none, 3 = severe), with a cumulative maximum score of 15. Scoring was performed blindly by two independent investigators. Scratching behavior was quantified by video analysis, and the number of scratching bouts was multiplied by 6 to obtain the final index. (**C**) Body weight (g) and (**D**) Daily food intake (g/day) were recorded throughout the experimental period to assess systemic effects of AD induction and treatment. (**E–F**) Assessment of immune organ hypertrophy. (**E**) Representative spleen images and spleen-to-body weight ratios (mg/g). (**F**) Representative inguinal lymph node images and corresponding lymph node-to-body weight ratios (mg/g). AD induction resulted in significant splenomegaly and lymphadenopathy, both of which were ameliorated by E-CME administration. Data are presented as mean ± SD (n = 8 per group). Statistical significance was determined using one-way ANOVA followed by Tukey’s post hoc test. #*p* < 0.05 vs. the control (Con) group; *p < 0.05 vs. the atopic dermatitis (AD) group.

As shown in Fig. 3A, the dorsal skin of mice in the AD group displayed severe dermatitis with visible signs of inflammation and epidermal damage. In contrast, the AD + E-CME and AD + Dermatop groups exhibited marked improvements, with attenuation of erythema, reduced scaling, and restored skin texture. These macroscopic observations were corroborated by quantitative dermatitis scoring (Fig. 3B), which assessed five clinical parameters on a 0–3 scale (maximum score = 15). The AD group recorded significantly elevated scores compared to all other groups (*p* < 0.01), while both treatment groups demonstrated a significant reduction in clinical severity (*p* < 0.05 vs. AD), with no significant difference between E-CME and Dermatop.

In addition to skin lesion assessments, systemic physiological parameters were monitored to evaluate the broader impact of treatment. As illustrated in Fig. 3C and 3D, the AD group experienced significant reductions in both body weight and daily food intake over the experimental period (*p* < 0.05 vs. CON and V), suggesting systemic stress or metabolic disturbance induced by chronic inflammation. Notably, E-CME treatment prevented these declines, maintaining body weight and feeding behavior at levels comparable to the control groups.

To further assess the systemic immunomodulatory effects of E-CME, spleen and lymph node hypertrophy were examined as indicators of immune activation. Representative images and weights of excised spleens are shown in Fig. 3E. Mice in the AD group exhibited significant splenomegaly compared to controls (*p* < 0.05), reflecting heightened systemic immune activity. Treatment with E-CME or Dermatop significantly ameliorated this enlargement, with spleen weights significantly lower than the AD group (*p* < 0.05), suggesting effective suppression of immune hyperresponsiveness.

Similarly, inguinal lymph node enlargement—a hallmark of peripheral immune activation—was pronounced in the AD group (Fig. 3F). Quantitative analysis revealed a significant increase in lymph node weight compared to the CON and V groups (*p* < 0.05), which was substantially reduced following E-CME or Dermatop administration (*p* < 0.05 vs. AD). Again, no significant difference was observed between the E-CME and Dermatop groups.

Collectively, these findings demonstrate that topical E-CME not only mitigates DNCB-induced cutaneous inflammation and clinical symptoms of AD but also exerts beneficial systemic effects by preserving physiological parameters and suppressing immune organ hypertrophy. The comparable efficacy to Dermatop suggests E-CME is a promising natural therapeutic candidate for the management of atopic dermatitis and associated systemic immune dysregulation.

### E-CME Modulates Systemic Immune Responses and Attenuates Oxidative Stress in AD Mice

**Fig. 4:**
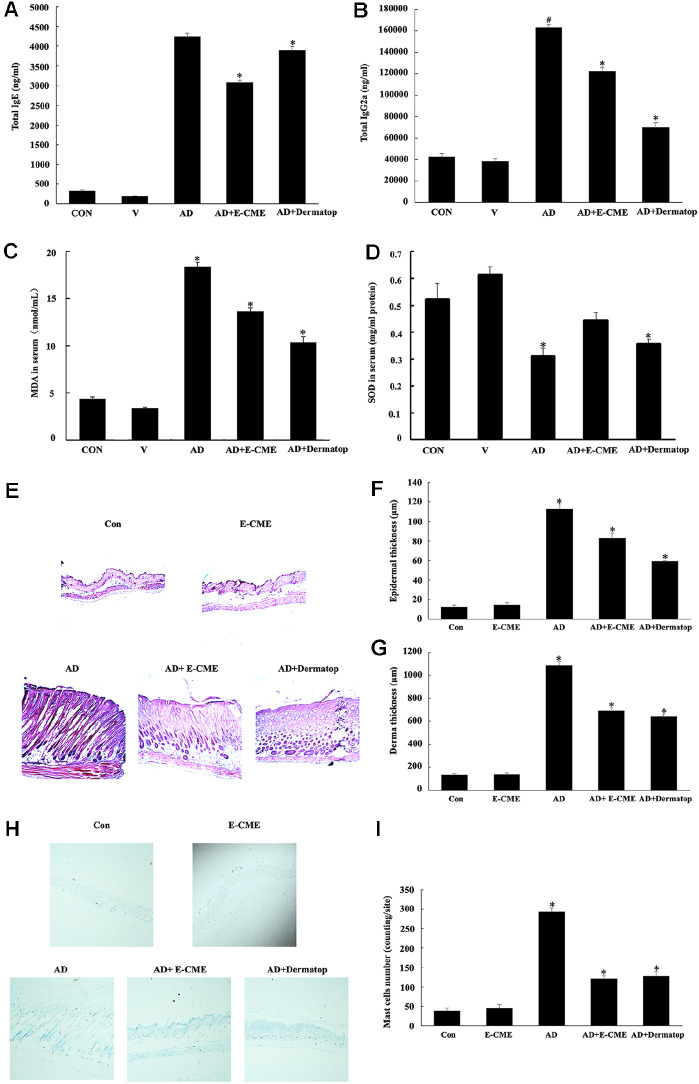
Effects of ethanol-extracted Chaga mushroom extract (E-CME) on systemic immune markers and histopathological features in a DNCB-induced atopic dermatitis (AD) mouse model. Female BALB/c mice (8 weeks old) were sensitized and challenged with 2,4-dinitrocholorobenzene (DNCB; 1% w/v in acetone-olive oil, 3:1, v/v) to induce AD-like lesions. E-CME (100 mg/kg) was topically applied thrice a week for 3 weeks and physiological saline served as vehicle control. (**A**) Serum levels of immunoglobulin E (IgE), immunoglobulin G2a (IgG2a), malondialdehyde (MDA), and superoxide dismutase (SOD) were measured using ELISA kits. DNCB-induced AD mice showed significantly elevated IgE and MDA levels, accompanied by reduced IgG2a and SOD, reflecting an enhanced Th2 response and oxidative stress both of which markedly reversed by E-CME treatment. (**B**) Representative hematoxylin and eosin (H&E) staining of dorsal skin sections (6 μm thickness; х 200 magnification) showing that AD mice exhibited pronounced epidermal hyperplasia, dermal thickening, and dense inflammatory cell infiltration, all attenuated by E-CME administration. (**C-D**) Histological quantification of epidermal thickness (**C**) and dermal thickness (**D**). Both were significantly thickened in AD mice and reduced by E-CME. **E–F**: Representative images of toluidine blue-stained skin sections (**E**; х400 magnification) and quantification of mast cell infiltration per site (**F**). Mast cell numbers were markedly increased in the AD group, indicating allergic inflammation, and were significantly decreased following E-CME treatment. Data are presented as mean ± SD (n = 8 per group). Statistical significance was determined by one-way ANOVA followed by Tukey’s post hoc test. #*p* < 0.05 vs. the control (Con) group; *p < 0.05 vs. the AD group.

To elucidate the systemic immunomodulatory and antioxidative effects of E-CME, serum levels of immunoglobulins and oxidative stress markers were measured in the DNCB-induced AD mouse model. As shown in Fig. 4A, DNCB application significantly elevated serum IgE levels, indicative of a Th2-skewed immune response (*p* < 0.05 vs. CON). E-CME administration effectively suppressed this IgE overproduction, achieving a 26.19% inhibition rate, which surpassed the Dermatop group (7.14%), suggesting a more pronounced anti-allergic effect. In contrast, serum IgG2a levels, which are typically associated with Th1 responses, were markedly reduced in the AD group and restored upon treatment. Notably, Dermatop induced a stronger IgG2a recovery (43.81% increase) compared to E-CME (24.07%). Oxidative stress was assessed by quantifying malondialdehyde (MDA) and superoxide dismutase (SOD) activity. MDA levels were significantly increased in the AD group (*p* < 0.05 vs. CON), reflecting enhanced lipid peroxidation and oxidative damage. E-CME treatment substantially decreased MDA concentrations (*p* < 0.05 vs. AD), whereas SOD activity was significantly augmented (*p* < 0.05 vs. CON), indicating restoration of antioxidant defenses. These results collectively suggest that E-CME not only modulates aberrant immune responses but also confers protection against oxidative stress in AD conditions.

Histopathological examination of dorsal skin sections was performed to evaluate tissue-level improvements following treatment. Hematoxylin and eosin (H&E) staining revealed that AD mice exhibited classical histological hallmarks of atopic dermatitis, including pronounced epidermal hyperplasia, marked dermal thickening, and dense infiltration of inflammatory cells such as lymphocytes and eosinophils (Fig. 4B). These pathological alterations were notably attenuated in both the AD + E-CME and AD + Dermatop groups, with no significant differences observed between them. Quantitative morphometric analysis (Fig. 4C and 4D) confirmed these findings. The AD group showed significant increases in both epidermal and dermal thickness (*p* < 0.05 vs. CON). E-CME treatment significantly reduced epidermal thickness (*p* < 0.05 vs. AD), and a similar trend was observed in dermal measurements, indicating its efficacy in reversing DNCB-induced skin hyperplasia and fibrosis. To further assess allergic inflammation, mast cell infiltration was evaluated using toluidine blue staining (Fig. 4E). AD mice exhibited a dramatic increase in dermal mast cell density compared to controls (*p* < 0.05), consistent with heightened hypersensitivity responses. Treatment with E-CME markedly suppressed mast cell infiltration (*p* < 0.05 vs. AD), as quantified in Fig. 4F, supporting its anti-allergic and anti-inflammatory properties. Taken together, these results demonstrate that E-CME not only normalizes systemic immunological and oxidative parameters but also effectively ameliorates histopathological abnormalities and immune cell infiltration in DNCB-induced AD lesions. The comparable efficacy of E-CME to Dermatop further underscores its therapeutic potential as a natural alternative for atopic dermatitis management.

### E-CME Attenuates AD-Associated Inflammation by Downregulating Multiple Cytokines

**Fig. 5:**
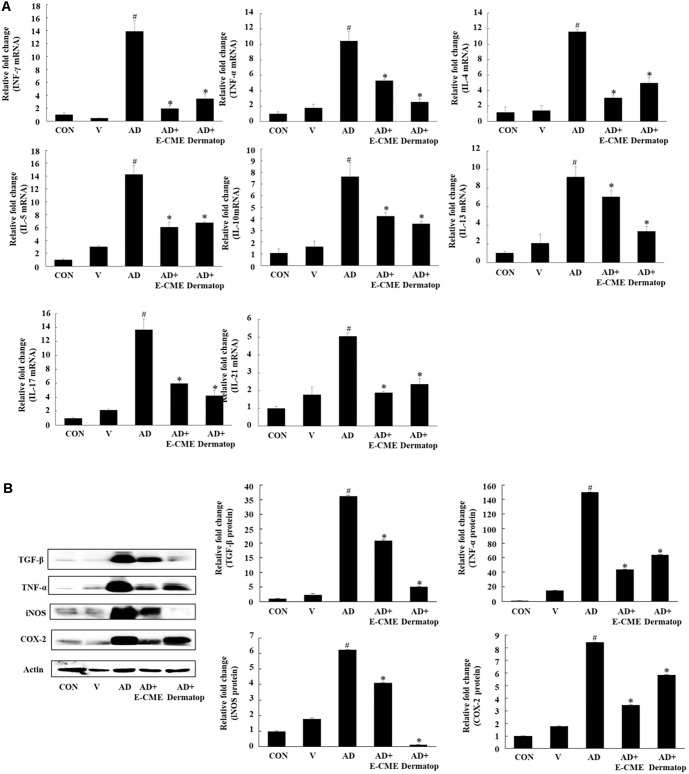
Effects of ethanol-extracted Chaga mushroom extract (E-CME) on pro- and anti-inflammatory mediators and MAPK signaling in a DNCB-induced atopic dermatitis (AD) murine model. (**A**) Quantitative real-time PCR analysis of mRNA expression levels of cytokines in dorsal skin tissues, including interferon-gamma (IFN-γ), tumor necrosis factor-alpha (TNF-α), interleukin (IL)-4, IL-5, IL-10, IL-13, IL-17, and IL-21. Expression levels were normalized to glyceraldehyde-3-phosphate dehydrogenase (GAPDH) and expressed as fold change relative to the untreated control (CON) group. (**B**) Western blot analysis of inflammatory mediators in skin tissue lysates, including transforming growth factor-beta (TGF-β), TNF-α, inducible nitric oxide synthase (iNOS), and cyclooxygenase-2 (COX-2). β-actin served as the internal loading control. Densitometric quantification of protein expression shown in panel B, presented as relative fold change compared with the CON group. Data are presented as mean ± SD (n = 8 per group). Statistical significance was determined by one-way ANOVA followed by Tukey’s post hoc test. #*p* < 0.05 vs. the control (Con) group; *p < 0.05 vs. the atopic dermatitis (AD) group.

To investigate the anti-inflammatory mechanism of E-CME, we first examined its effects on cytokine expression levels in dorsal skin tissues. As shown in Fig. 5A, qPCR analysis revealed that DNCB exposure significantly upregulated the mRNA levels of Th1 (IFN-γ, TNF-α), Th2 (IL-4, IL-5, IL-10, IL-13), and Th17 (IL-17, IL-21) cytokines compared to the control group (#*p* < 0.05). Treatment with E-CME led to a marked reduction in the expression of all these cytokines (*p < 0.05 vs. AD), indicating its broad immunomodulatory activity and potential to counteract the aberrant immune responses associated with atopic dermatitis (AD).

Fig. 5B further validated these findings at the protein level using Western blot analysis. The AD group exhibited a substantial increase in the protein levels of TNF-α, TGF-β, iNOS, and COX-2, all of which are key mediators of skin inflammation. Notably, E-CME treatment significantly suppressed the expression of these inflammatory proteins to levels comparable to the Dermatop-treated group. Importantly, E-CME did not induce inflammatory cytokine expression in non-DNCB-treated mice, confirming its selective anti-inflammatory activity without disrupting physiological immune homeostasis.

### E-CME Suppresses MAPK Pathway Activation in DNCB-Induced AD

**Fig. 6:**
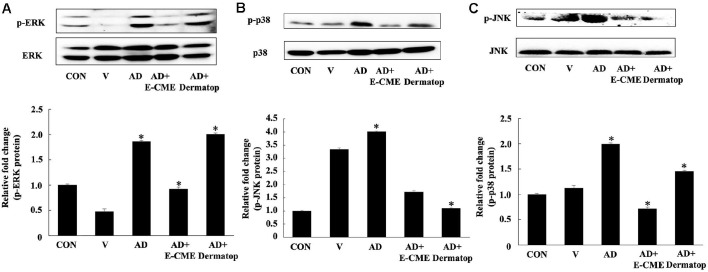
Inhibitory effects of ethanol-extracted Chaga mushroom extract (E-CME) on MAPK pathway activation in DNCB-induced atopic dermatitis (AD) mice. Western blot analysis of dorsal skin tissues was conducted to evaluate phosphorylation levels of key mitogen-activated protein kinases (MAPKs), including (**A**) extracellular signal-regulated kinase ½ (ERK1/2), (**B**) p38 MAPK, and (**C**) c-Jun N-terminal kinase (JNK). Both phosphorylated and total protein levels were detected and β-actin served as internal loading control. Densitometric analysis was used to quantify protein bands, and phosphorylation levels were normalized to total MAPK expression. E-CME treatment markedly suppressed DNCB-induced phosphorylation of ERK1/2, p38, and JNK indicating suppression of MAPK suppression. E-CME treatment markedly attenuated DNCB-induced phosphorylation of ERK1/2, p38, and JNK, indicating suppression of MAPK pathway activation. Data are presented as mean ± SD (n = 8 per group). Statistical significance was determined by one-way ANOVA followed by Tukey’s post hoc test. #*p* < 0.05 vs. the control (Con) group; *p < 0.05 vs. the atopic dermatitis (AD) group.

To further elucidate the molecular mechanisms underlying the anti-inflammatory effects of E-CME, we assessed the phosphorylation status of mitogen-activated protein kinases (MAPKs), which are central mediators of inflammatory signaling. As illustrated in Fig. 6, DNCB exposure led to a significant increase in the phosphorylation of ERK1/2, p38, and JNK, indicating robust activation of MAPK signaling in the AD group (#*p* < 0.05 vs. Con). E-CME treatment markedly reduced the phosphorylation levels of all three MAPKs (*p < 0.05 vs. AD), suggesting that its anti-inflammatory effects are mediated, at least in part, through suppression of MAPK pathway activation.

It is worth noting that E-CME did not significantly alter the total expression levels of these MAPKs, highlighting that its effect is specific to inhibition of phosphorylation rather than overall protein abundance. In comparison, the Dermatop-treated group exhibited only partial suppression of MAPK activation, underscoring the superior efficacy of E-CME in targeting intracellular inflammatory signaling pathways.

## Discussion

AD is a chronic, relapsing inflammatory skin disorder driven by complex interactions among genetic predisposition, skin barrier dysfunction, immune dysregulation, and environmental triggers. In particular, oxidative stress and the imbalance of Th1/Th2/Th17 immune responses are considered pivotal in the pathogenesis of AD [12, 25]. Consequently, natural compounds with both antioxidant and immunomodulatory activities are increasingly being investigated as potential therapeutic agents for AD [26].

CM, being a long-used traditional medicine, is rich in diverse bioactive compounds such as polysaccharides, glucans, triterpenoids (including lupeol, lanosterol, and inotodiol), melanin, polyphenol, phelligridin D, 3,4-dihydroxybenzalacetone, caffeic acid, inonoblin B, terpenes, betulin, BA, sterols, ergosterols peroxide, and trametenolic acid. A qualitative analysis of CM secondary metabolites has identified 111 phenolic, 63 fatty/aromatic acid, and 108 terpenoid constituents [27]. Hwang *et al*., 2019 reports 5.88% total glucans, 24.3 mg UAE (ursolic acid equivalent)/g triterpenoids, and 204 mg GAE (gallic acid equivalent)/g total phenolic compounds in CM, though at different extraction conditions [28]. The broad range of constituents in CM collectively offer diverse biological activities, including anticancer, anti-inflammatory, antiviral, antioxidant, immunomodulatory, hypoglycemic, hypolipidemic, and hepatoprotective effects [3, 14, 29-31]. In addition, CM bioactive compounds have been reported to regulate some gene expression and confer anti-apoptotic, anti-cell proliferative, and cytoprotective activities against heavy metal-induced cellular damage [3]. In the current study, the combined action of CM polysaccharides, glucans, triterpenoids (including BA and betulin), melanin, and phenolic compounds is proposed to contribute to its observed anti-inflammatory, antioxidant, and immunoregulatory effects. However, limited studies have investigated its therapeutic efficacy in murine models of AD. To address this gap, we evaluated the protective effects of ethanol extract of Chaga mushroom (E-CME) in a DNCB/DFE-induced AD mouse model and *in vitro* cell systems.

Betulin and BA have been studied in some dermal pathophysiological conditions, demonstrating anti-inflammatory, antioxidant, wound healing, and skin-barrier protective roles. They help reduce skin inflammation and oxidative stress, mitigate itching and allergic responses, promote wound healing and support overall skin barrier integrity [24, 32, 33]. During this study, the levels of BA and betulin in E-CME were assessed, as these compounds are therapeutically crucial for managing AD. Betulin is a well-known ingredient in traditional birch-derived medicinal preparations owing to its antioxidant and anti-inflammatory properties. Mechanistically, betulin inhibit NF-κB activation by suppressing IKK phosphorylation, limiting IκBα degradation and p65 nuclear translocation, thereby reducing expression of downstream mediators. This mechanism is associated with reducing cytokine release, leukocyte recruitment, and barrier disruptive gene expression that indicates AD inflammation [34, 35].

BA has been already studied in inflammatory skin disease models such as psoriasis and eczema. In an imiquimod-induced psoriasis model, BA reduced epidermal thickness, T-cell infiltration, IL-17A producing CD4^+^ and γδ T-cells, and expression of IL-17A, IL-6, TNF-α, while suppressing NF-κB signaling and IL-10, indicating potent Th17/Th1 modulation. These mechanisms suggest that BA could theoretically ameliorate lipid barrier and inflammatory disturbance observed during AD [33, 36]. However, the improvements observed in this study cannot be solely attributed to BA or betulin, since isolated betulin or BA were not used. Moreover, betulin or BA have not yet been systematically characterized in classical AD models, representing an important avenue for future research.

### E-CME Demonstrated Significant Antioxidant Potential

Oxidative stress is a known contributor to AD, promoting epidermal damage and immune cell activation [34]. In this study, E-CME exhibited strong antioxidant capacity as evidenced by multiple *in vitro* assays, including DPPH, H_2_O_2_ radical scavenging, metal chelation, and FRAP assays (Fig. 2). These findings are consistent with previous reports that Chaga extracts are rich in polyphenols and other redox-active compounds capable of scavenging free radicals and modulating oxidative enzymes [37, 38].

*In vivo*, treatment with E-CME significantly reduced malondialdehyde (MDA) levels and increased superoxide dismutase (SOD) activity, further confirming its systemic antioxidant effects (Fig. 4C and 4D). Oxidative stress reduction may, in turn, attenuate the production of inflammatory cytokines and help restore epidermal barrier function [39].

### E-CME Suppressed Mast Cell Degranulation and TNF-α Expression *In Vitro*

Mast cells play a central role in allergic inflammation by releasing histamine, cytokines, and proteolytic enzymes upon activation. Using HaCaT keratinocytes and RBL-2H3 mast cells, we found that E-CME markedly reduced TNF-α expression and inhibited β-hexosaminidase release in a dose-dependent manner (Figs. 4 and 5). Since β-hexosaminidase is a classical marker of mast cell degranulation [40], these results suggest that E-CME stabilizes mast cells and downregulates pro-inflammatory mediators. These *in vitro* findings are consistent with *in vivo* data showing reduced mast cell infiltration in E-CME–treated skin lesions.

### E-CME Alleviated Clinical and Histological Manifestations of AD

Topical application of E-CME significantly attenuated the overall clinical severity of AD in DNCB/DFE-induced mice, as evidenced by significant reduction of erythema, scaling, and edema (Fig. 5), key clinical signs reflecting epidermal barrier disruption and dermal inflammation. These improvements in visible key pathology are corresponded with the mitigation of systematic symptoms such as DNCB-induced weight loss and anorexia, indicating an overall reduction in the disease burden and systemic inflammation (Fig. 5A and 5B). Histologically, E-CME reduced epidermal hyperplasia and dermal thickening which are hallmarks of skin remodeling and chronic inflammation in AD. Along with that, it also diminished the infiltration of inflammatory cells, reflecting restoration of structural integrity and immune homeostasis [41-43].

In addition, E-CME treatment normalized spleen and lymph node enlargement (Fig. 6), mechanical indicators of systemic immune activation and lymphoproliferation commonly concomitant with AD. These effects align with the previous studies that emphasize spleen hypertrophy in AD models. Together these findings highlighted the ability of E-CME to alleviate both the mechanical skin damage and systemic immunopathological features characteristic of AD [44, 45]. Nevertheless, this study was limited to short-term treatment in a murine model, and long-term safety or efficacy data remains to be established. Additionally, the mechanistic findings were derived from immortalized cell lines rather than primary keratinocytes or immune cells, warranting further validation using physiologically relevant models. Another limitation of this study is that the residual ethanol content in the E-CME was not quantitatively analyzed. Although no signs of local irritation or adverse effects were observed during topical application, further studies should include precise quantification of residual solvents to ensure compliance with safety standards and to strengthen the transitional relevance of findings.

### E-CME Modulated Immunoglobulin Levels and T Helper Cell Responses

Elevated serum IgE, a hallmark biomarker of allergic inflammation in AD, reflects an overexpressive Th2 immune response primarily driven by cytokines such as IL-4 and IL-5 [46]. In this study, E-CME significantly suppressed serum IgE and IgG2a levels, which demonstrates its potential for rebalancing of the Th1/Th2 axis toward immune homeostasis. Importantly, E-CME showed greater efficacy in reducing IgE compared to the positive control (Dermatop), emphasizing its potential as an effective alternative therapeutic agent.

Beyond the classical Th1/Th2 paradigm, recent evidence implicates Th17 cells and their associated cytokines in chronic or severe AD pathogenesis [4, 47, 48]. Our qPCR and Western blot analyses revealed that E-CME exerts broad immunomodulatory effects by downregulating cytokines across multiple T-cell subsets: Th1 (IFN-γ, TNF-α), Th2 (IL-4, IL-5, IL-10, IL-13), and Th17 (IL-17, IL-21), as well as inflammatory mediators such as iNOS, COX-2, and TGF-β. These effects imply that E-CME targets multiple signaling pathways integrated with immune cell activation and skin inflammation, including pathways responsible for cytokine production, mast cell activation, and recruitment of inflammatory cells. These comprehensive immunomodulatory actions contribute to the pronounced therapeutic efficacy of E-CME for disrupting the chronic inflammatory cycle and restoring skin integrity.

### E-CME Modulated MAPK Signaling

The mitogen-activated protein kinase (MAPK) pathway, including ERK, p38, and JNK, is critically involved in regulating cytokine production, immune cell activation, and inflammatory responses during allergic diseases such as AD [49]. In the present study, E-CME significantly suppressed phosphorylation of ERK1/2, p38, and JNK in skin tissue, highlighting the ability to directly inhibit pro-inflammatory MAPK signaling cascades that drive keratinocyte activation and inflammatory cell infiltration in AD. Notably, the positive control Dermatop did not exert significant effects on MAPK phosphorylation, suggesting a potent mechanistic profile for E-CME in modulating intracellular signaling [50].

All in all, these findings deepen the understanding of how E-CME reduced AD pathology, not only by modulating cytokine expression and immune cell function but also by targeting key intracellular signaling nodes that lead to inflammatory responses and tissue remodeling. These results align with previous studies demonstrating that MAPK inhibition attenuates keratinocyte hyperproliferation and cytokine secretion in AD models. Future research on precise molecular targets affected by E-CME and the signaling modulation dynamics during E-CME treatment will help to further deepen the understanding of E-CME against AD.

## Conclusion

Collectively, this study provides the first comprehensive evidence that ethanol extract of *Inonotus obliquus* (E-CME) effectively alleviates AD-like symptoms in a murine model through synergetic antioxidant, anti-inflammatory, and immunomodulatory mechanisms. E-CME significantly reduced oxidative stress, serum immunoglobulin levels, mast cell activation, and the expression of key inflammatory cytokines, effects that were mediated, at least in part, by the inhibition of MAPK phosphorylation. Given its potent bioactivity and minimal side effects, E-CME demonstrates strong potential as a novel natural therapeutic agent for the management of atopic dermatitis.

## Supplemental Materials

Supplementary data for this paper are available on-line only at http://jmb.or.kr.



## Figures and Tables

**Table 1:** List of primers for real-time PCR.

Name	Forward	Reverse
GAPDH	5'-CATGG CCTTCCGTGTT CCTA-3'	5'-TGTCATCATACTTGGCAGGTTTCT-3'
TNF-α	5'-AAGCCTGTAGCCCACGTCGTA-3'	5'-GGCACCACTAGTTGGTTGTCTTTG-3'
IL-4	5'-TCTCGAAtGTACCAGGAGCCATATC-3'	5'-AGCACCTTG GAAGCCCTACAGA-3'
IL-5	5'-ACAGGAGAAGGGACGCCAT-3'	5'-GAAGCCCGTACAGACGAGCTCA-3'
IL-17	5'-TCCCCTCTGTCATCTGGGAAG-3'	5'-CTCGACCCTGAAAGTGAAGG-3'
IL-6	5'-CCACTTCACAAGTCGGAGGCTTA-3'	5'-GCAAGTGCATCATCGTTGTTCATAC-3'
IL-10	5'-TCAGCTGTGTCTGGGGCCACT-3'	5'-TTATGAGTAGGGACAGGAAGCCTCA-3'
IL-13	5'-GCA ACA TCA ACA GGA CCA GA-3'	5'-GTC AGG GAA TCC AGG GCT AC-3'
IFN-γ	5'-TCAAGTGGCATAGATGTGGAAGAA-3'	5'-TGGCTCTGCAGGATTTTCATG-3'
IL-21	5'-GCAGGGAGGAGACAGAACACA-3'	5'-GAGTTCCTCACTTCCGTGGT-3'

**Table 2:** E-CME extraction yields.

Batch number	Dry weight of raw materials (g)	Extraction Solvent (ml, Ethanol)	Weight of extract (mg)	Extraction yield (%)
1	9.85	100	344.8	3.5
2	10.12	100	374.4	3.7
3	9.97	100	376.9	3.78
