# Supplementary material for: Chaga Mushroom (Inonotus obliquus) Attenuates DNCB-Induced Atopic Dermatitis by Modulating Oxidative Stress and Cytokine Expression
Source: J Microbiol Biotechnol. 2026 Jan 22;36:e2510032. doi: 10.4014/jmb.2510.10032 (PMC12861731; doi:10.4014/jmb.2510.10032)
Supplement: Supplementary file 1 [file jmb-36-e2510032-supple.zip › jmb-36-e2510032-supple1.pdf]

J. Microbiol. Biotechnol. 2026. 36: e2510032
https://doi.org/10.4014/jmb.2510.10032

TNF-α

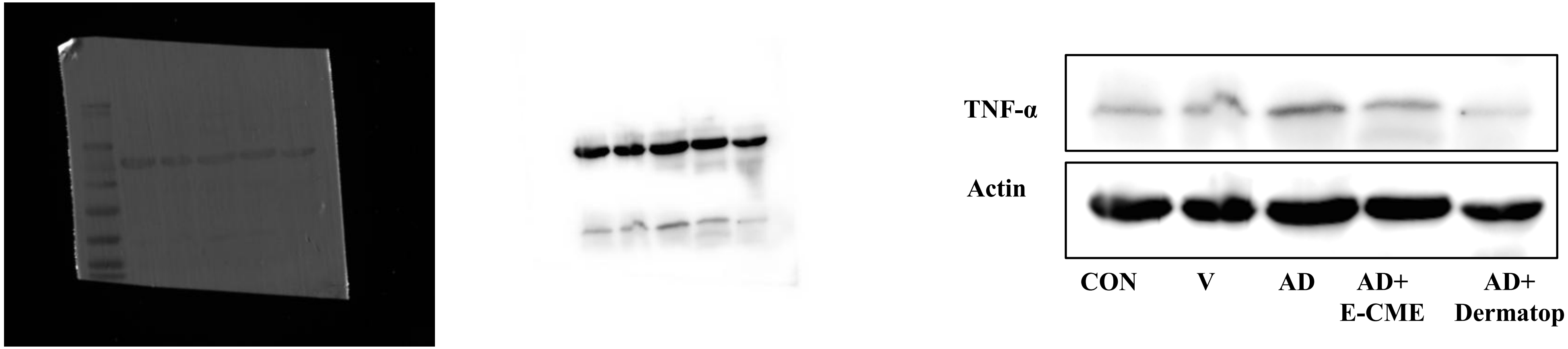

COX-2

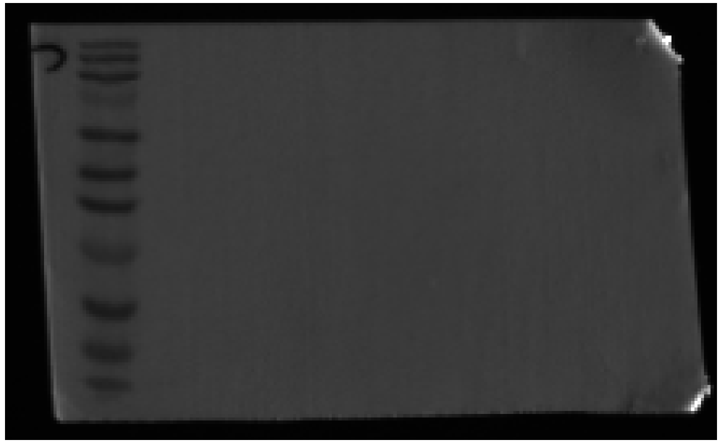

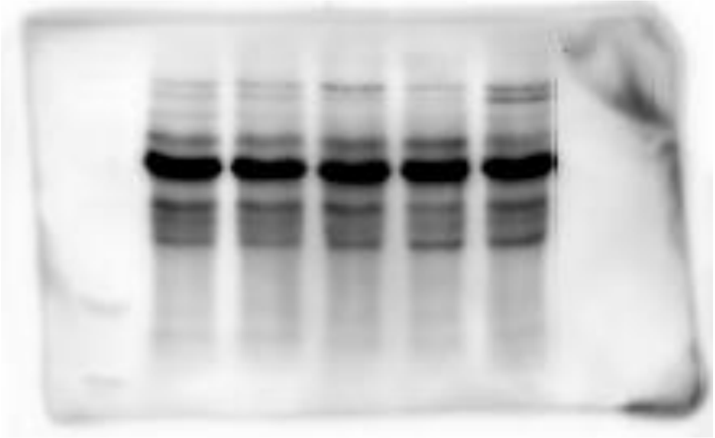

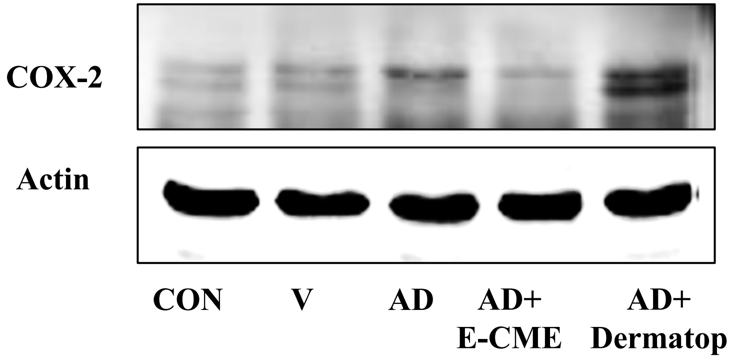

TGF-β

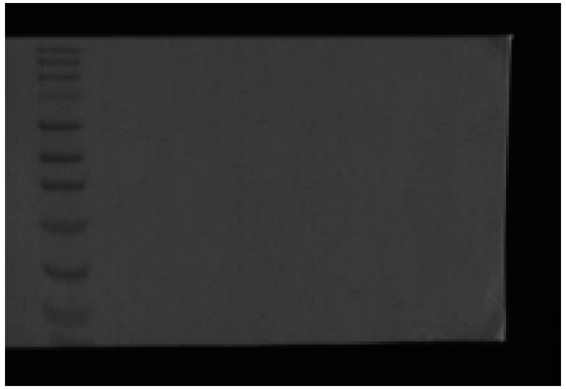

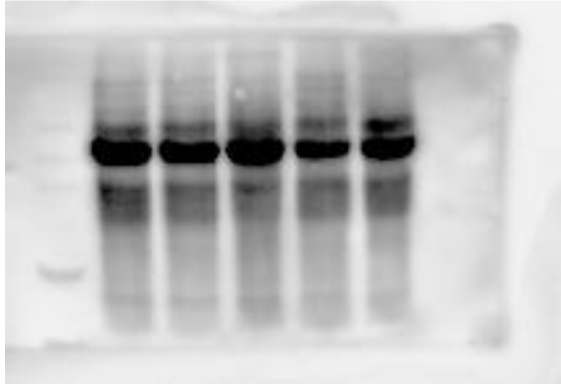

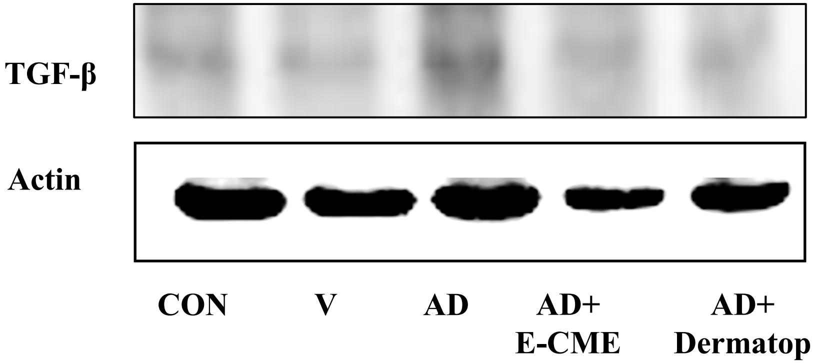

iNOS

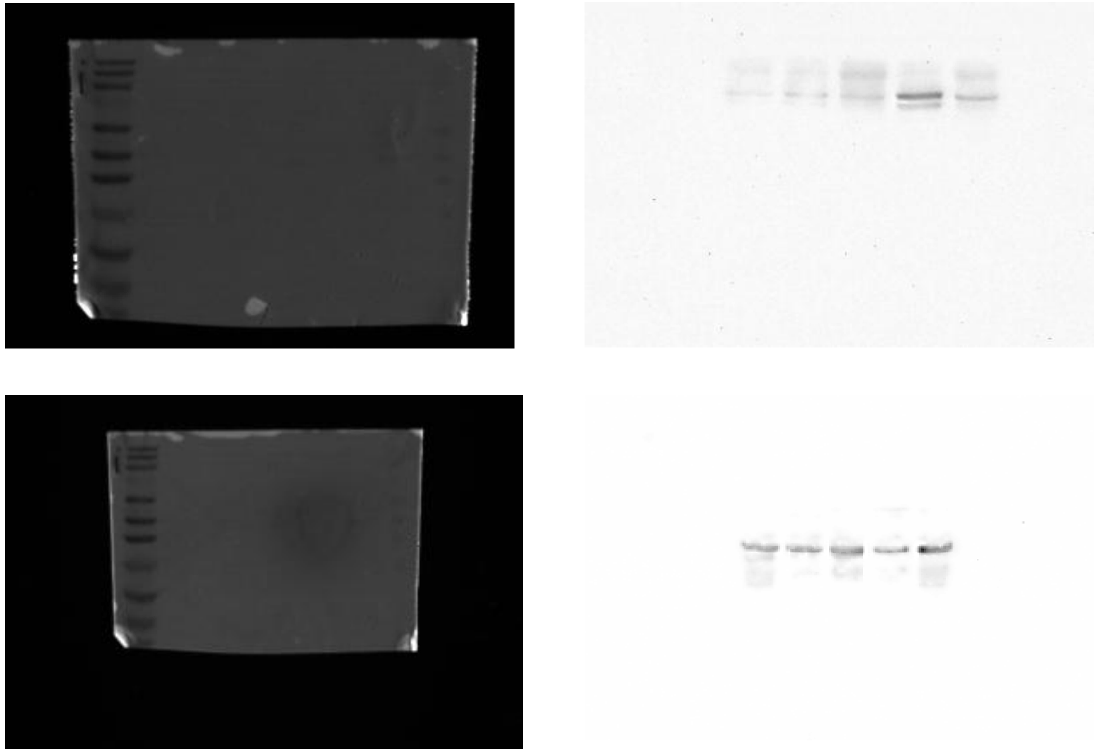

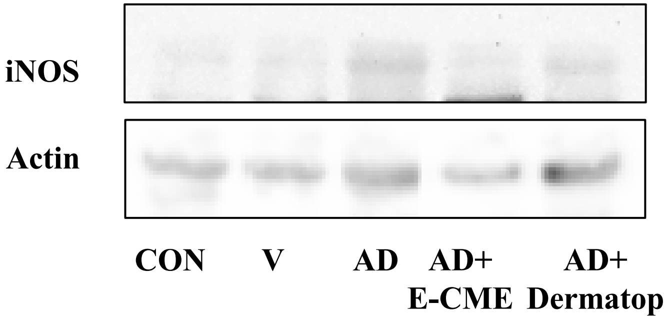

JNK

Actin

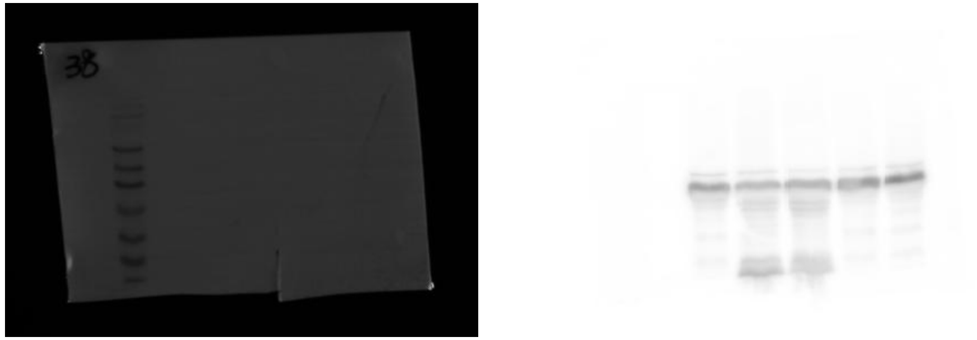

JNK

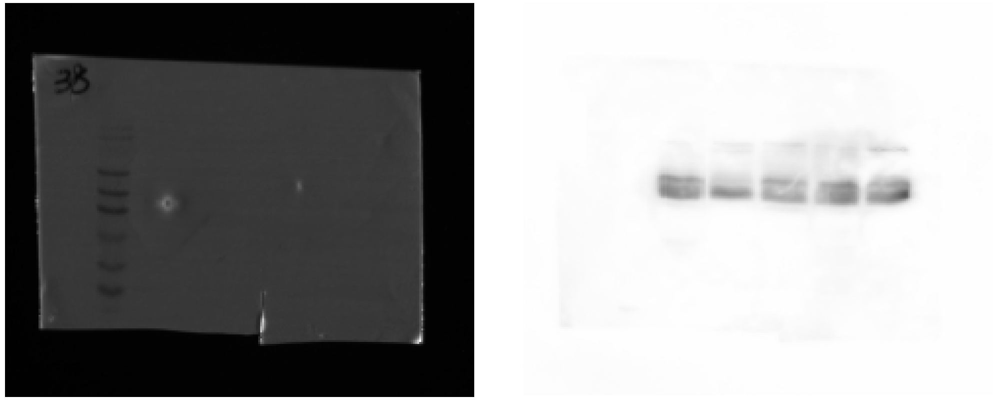

p-JNK

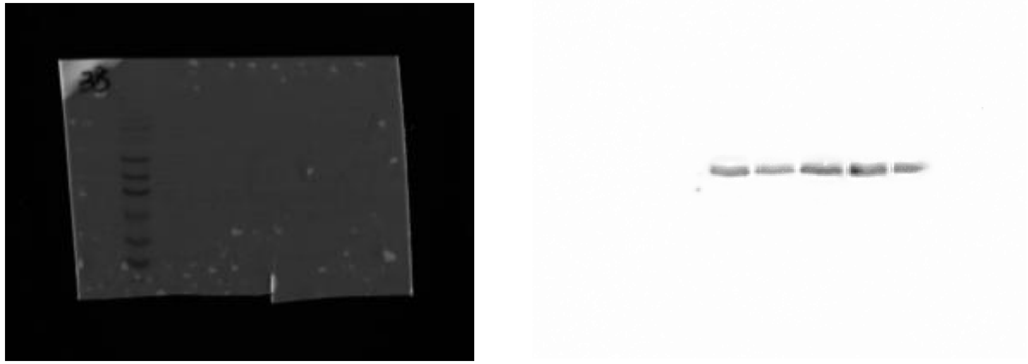

p-JNK

JNK

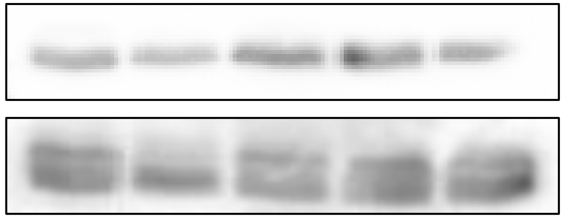

CON V AD AD+ E-CME AD+ Dermatop

ERK

GAPDH

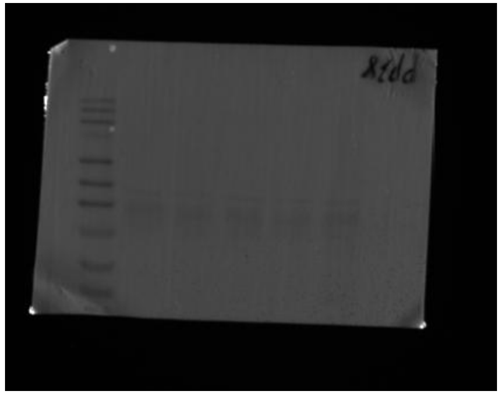

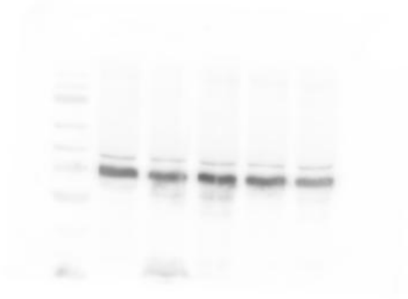

p-ERK

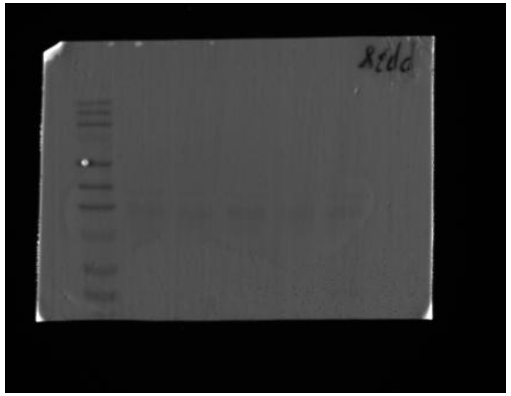

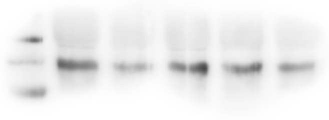

ERK

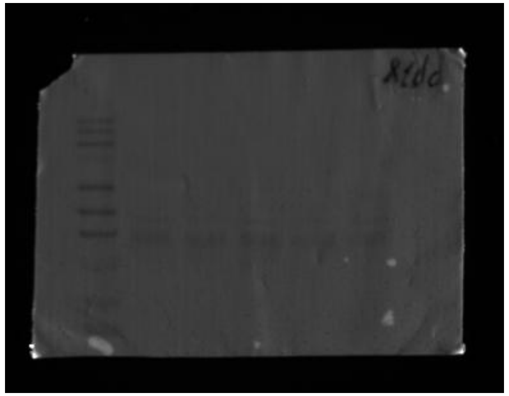

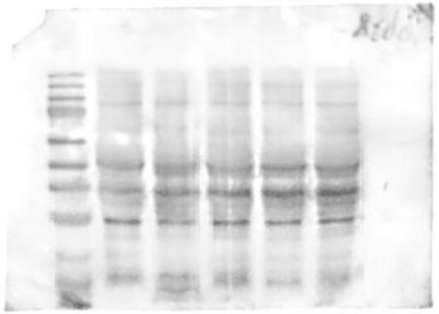

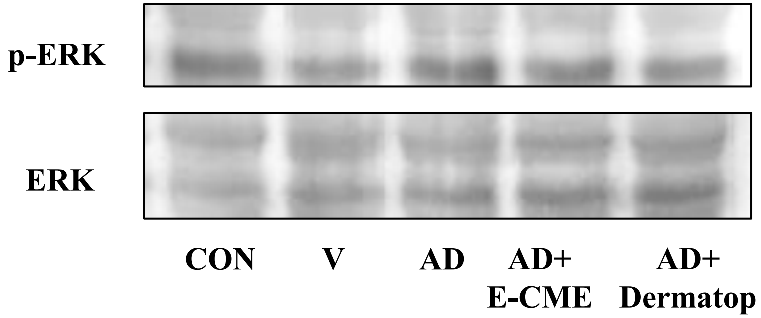

p38

p38

GAPDH

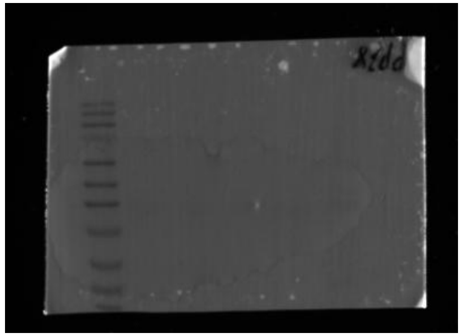

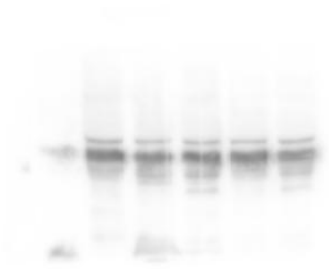

p-p38

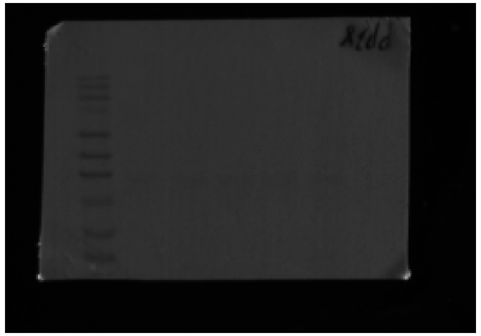

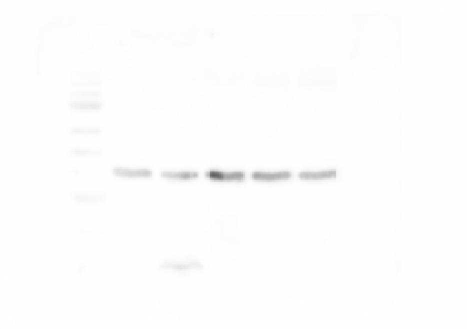

p-p38

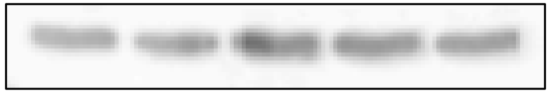

p38

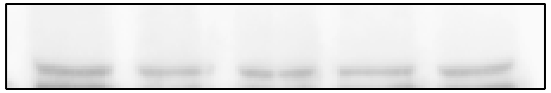

CON V AD AD+ E-CME AD+ Dermatop

Please consider that,
1. Some samples were stored at -80°C for 18 months. For target proteins with relatively low stability, minor degradation or conformational changes may have occurred, which has slightly impaired the antibody binding efficiency.
2. During whole-membrane development in Western blot assays, the internal reference protein exhibits high sensitivity, and its signal preferentially occupies the signal response range. This phenomenon suppresses the signals of low-abundance target proteins, thereby resulting in fainter target bands.
3. Reasonable errors inherent in biological experiments—such as batch-to-batch titer variations of reagents and minor deviations in instrument calibration—have not altered the core trends of the study.
