# Supplementary material for: Chaga Mushroom (Inonotus obliquus) Attenuates DNCB-Induced Atopic Dermatitis by Modulating Oxidative Stress and Cytokine Expression
Source: J Microbiol Biotechnol. 2026 Jan 22;36:e2510032. doi: 10.4014/jmb.2510.10032 (PMC12861731; doi:10.4014/jmb.2510.10032)
Supplement: Supplementary file 1 [file jmb-36-e2510032-supple.zip › jmb-36-e2510032-supple2.pdf]

J. Microbiol. Biotechnol. 2026. 36: e2510032

https://doi.org/10.4014/jmb.2510.10032

Supplementary Figures

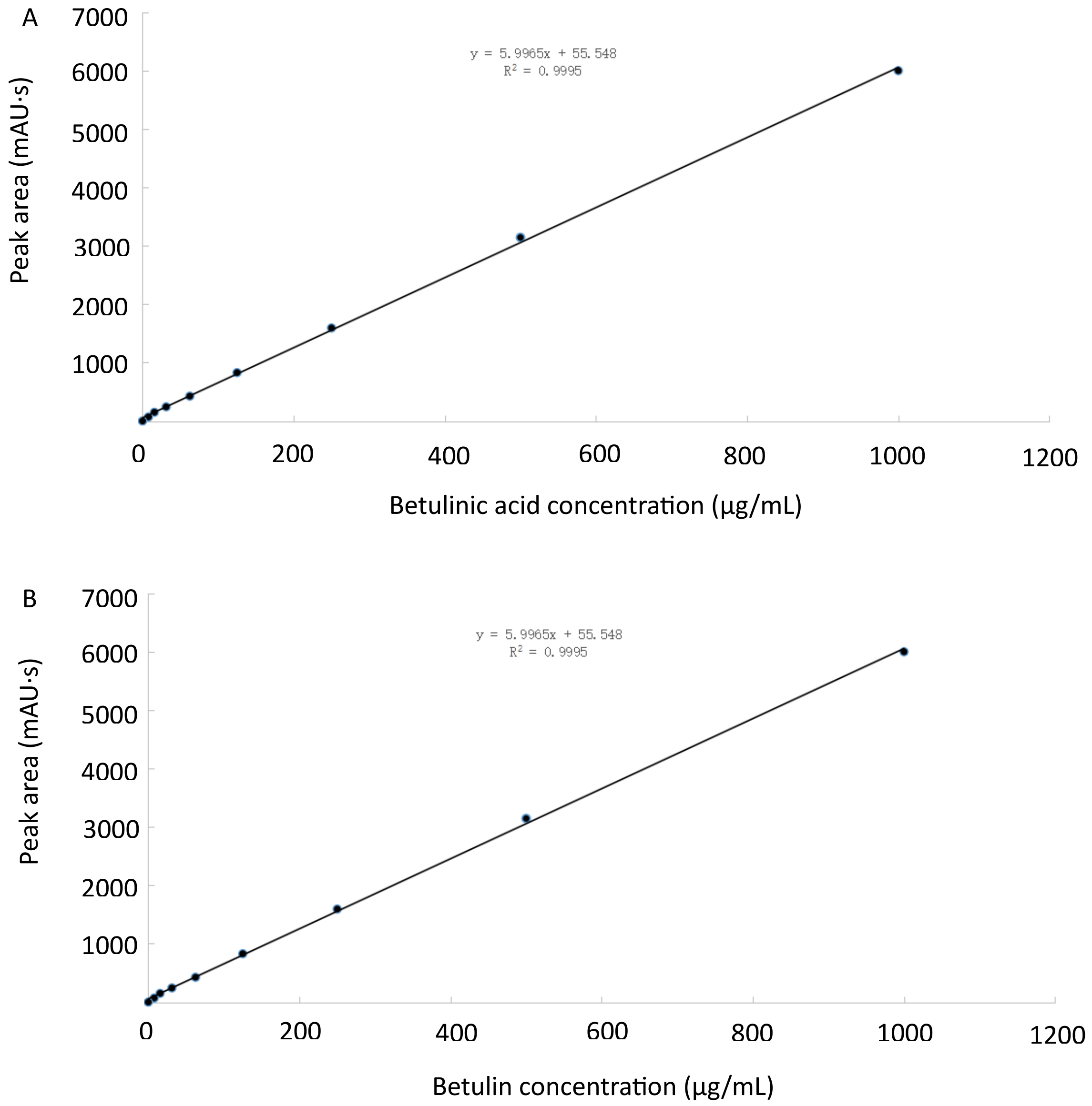

Fig. S1. Standard calibration curves for quantifying betulinic acid betulin in ethanol-extracted Chaga mushroom extract (E-CME): (A) Betulinic acid and (B) Betulin.

A

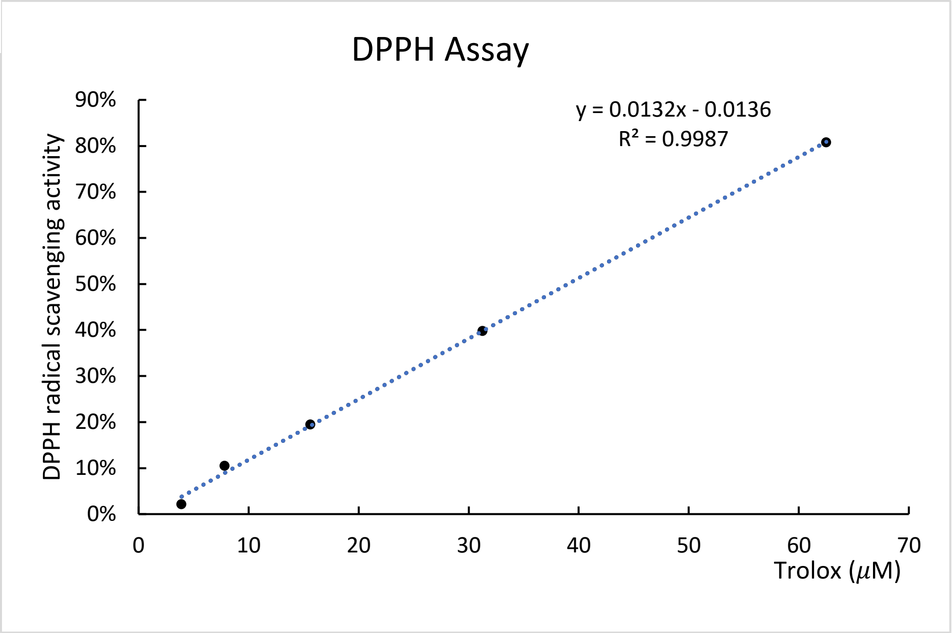

B

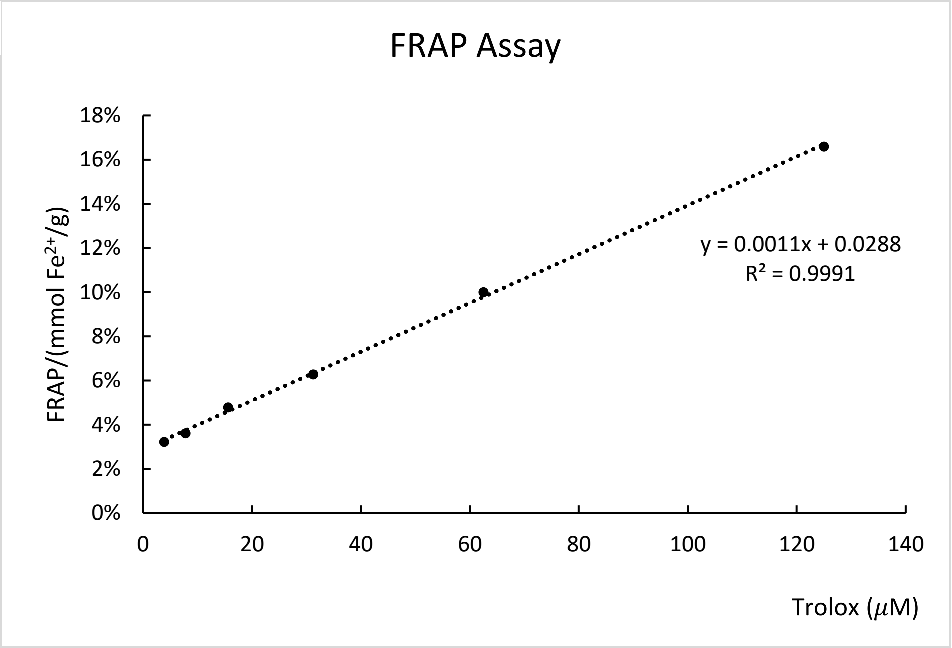

Fig. S2. Standard calibration curves for determining the antioxidant activity of ethanol-extracted Chaga mushroom extract (E-CME) using different assays: (A) DPPH assay and **(B)** FRAP assay. Trolox was used as the positive control. Standard calibration curves were generated by plotting percent inhibition against Trolox concentration.
